# Role of T3SS-1 SipD Protein in Protecting Mice against Non-typhoidal *Salmonella* Typhimurium

**DOI:** 10.1371/journal.pntd.0005207

**Published:** 2016-12-19

**Authors:** Bakhos Jneid, Karine Moreau, Marc Plaisance, Audrey Rouaix, Julie Dano, Stéphanie Simon

**Affiliations:** Service de Pharmacologie et Immunoanalyse (SPI), CEA, INRA, Université Paris-Saclay, Gif-sur-Yvette, France; Massachusetts General Hospital, UNITED STATES

## Abstract

**Background:**

*Salmonella enterica* species are enteric pathogens that cause severe diseases ranging from self-limiting gastroenteritis to enteric fever and sepsis in humans. These infectious diseases are still the major cause of morbidity and mortality in low-income countries, especially in children younger than 5 years and immunocompromised adults. Vaccines targeting typhoidal diseases are already marketed, but none protect against non-typhoidal *Salmonella*. The existence of multiple non-typhoidal *Salmonella* serotypes as well as emerging antibiotic resistance highlight the need for development of a broad-spectrum protective vaccine. All *Salmonella* spp. utilize two type III Secretion Systems (T3SS 1 and 2) to initiate infection, allow replication in phagocytic cells and induce systemic disease. T3SS-1, which is essential to invade epithelial cells and cross the barrier, forms an extracellular needle and syringe necessary to inject effector proteins into the host cell. PrgI and SipD form, respectively, the T3SS-1 needle and the tip complex at the top of the needle. Because they are common and highly conserved in all virulent *Salmonella* spp., they might be ideal candidate antigens for a subunit-based, broad-spectrum vaccine.

**Principal Findings:**

We investigated the immunogenicity and protective efficacy of PrgI and SipD administered by subcutaneous, intranasal and oral routes, alone or combined, in a mouse model of *Salmonella* intestinal challenge. Robust IgG (in all immunization routes) and IgA (in intranasal and oral immunization routes) antibody responses were induced against both proteins, particularly SipD. Mice orally immunized with SipD alone or SipD combined with PrgI were protected against lethal intestinal challenge with *Salmonella* Typhimurium (100 Lethal Dose 50%) depending on antigen, route and adjuvant.

**Conclusions and Significance:**

*Salmonella* T3SS SipD is a promising antigen for the development of a protective *Salmonella* vaccine, and could be developed for vaccination in tropical endemic areas to control infant mortality.

## Author Summary

*Salmonella* are bacteria responsible for a high global burden of invasive diseases, especially in South and South-East Asia (mainly enteric fever due to *Salmonella* Typhi) and sub-Saharan Africa (mainly invasive Non-Typhoidal *Salmonella*, iNTS). This iNTS disease has emerged as a prominent cause of systemic infection in children and immunocompromised African adults, with an associated case fatality of 20–25%. Because licensed vaccines only protect against enteric fever, there is a crucial need to develop a new broad-spectrum vaccine effective against enteric fever and iNTS that can be administered safely to children under 2 years old. The virulence of *Salmonella* depends on two type III secretion systems (T3SS-1 and T3SS-2) necessary for invasion, replication, intracellular survival and dissemination of the bacteria. Two structural proteins of T3SS-1 (essential for crossing the epithelial barrier) are highly conserved among *Salmonella* spp. and might be good candidates for a broad-spectrum vaccine. The current study describes the protective effect elicited by these proteins in a murine model. A specific immune response was generated against our antigens and provided protection against *Salmonella* Typhimurium oral infection. Such a candidate vaccine offers promising perspectives to control *Salmonella* diseases.

## Introduction

Salmonellae are members of the Enterobacteriaceae family, a large group of Gram-negative bacteria [1]. While consisting of only two species (*enterica* and *bongori*), there are a multiplicity of subspecies among which *enterica* spp. represent 99% of *Salmonella* infections in warm-blooded animals and humans [2–3]. The *Salmonella* that induce human diseases are divided into typhoidal serotypes (*S*. Typhi and *S*. Paratyphi A and B) and thousands of non-typhoidal serotypes [4]. While *Salmonella* infections are usually responsible for a self-limiting gastroenteritis or a relatively well controlled typhoid fever in healthy humans of high-income countries [5], they remain a serious health hazard in Asian and African countries, where they manifest as invasive illnesses associated with high morbidity and mortality rates [6]: enteric fever caused by Typhoidal *Salmonella* and mainly found in South and South-East Asia and invasive Non-Typhoidal *Salmonella* (iNTS) characterized by severe extra-intestinal invasive bacteremia in sub-Saharan Africa [7–8].

Currently, three types of *Salmonella* vaccines are licensed: the oral live attenuated *Salmonella* Typhi Ty21a vaccine and the parenteral Vi capsular polysaccharide antigen, either unconjugated or conjugated to tetanus toxin [9–12]. However, all of them target *S*. Typhi and are not cross-protective against other *Salmonella* serovars, except for slight protection afforded by the Ty21a vaccine against *S*. Paratyphi B (which represents a minority of cases among all paratyphoid cases) [13]. None of them are protective against non-typhoidal *Salmonella*. The multiplicity of *Salmonella* serovars [14], the high global burden of the disease as well as the emergence of strains resistant to anti-microbial drugs [15–18] make the development of an effective broad-spectrum *Salmonella* vaccine even more urgent. Thus, efforts to develop a multivalent vaccine that targets the different serovars (*S*. Typhi, *S*. Paratyphi, *S*. Typhimurium, *S*. Enteritidis and *S*. Choleraesuis) are needed to control invasive *Salmonella* infections worldwide [19].

*Salmonella*, as facultative intracellular bacteria, are found within a variety of phagocytic and non-phagocytic cells *in vivo* [20]. Following intestinal adherence, invasive *Salmonella* bacteria preferentially enter microfold (M) cells and transport them to lymphoid cells in the underlying Peyer’s Patches (PPs) [21]. *Salmonella* can also induce its internalization in non-phagocytic enterocytes and actively invade them through its virulence-associated type 3 secretion system encoded by Salmonella Pathogenicity Island 1 (SPI-1) [22]. After crossing the intestinal barrier at the site of PPs, the bacteria are taken up by phagocytic immune cells like macrophages and dendritic cells. Once phagocytosed, *Salmonella* replicate within a modified phagosome known as the *Salmonella*-containing vacuole (SCV) in the cytoplasm [23–24]. A second type 3 secretion system encoded by Salmonella Pathogenicity Island 2 (SPI-2) seems to play a crucial role in this replication process and in survival within macrophages and consequently in systemic virulence [25]. However, there is increasing evidence that the so-called chronological and localized partitions of SPI-1 and SPI-2 T3SS roles become less and less clear and that both T3SS-1 and T3SS-2 contribute to multiple stages of pathogenesis [26–27].

Type 3 secretion systems (T3SSs) or injectisomes are bacterial macromolecular organelles that are involved in the pathogenesis of many important human, animal and plant diseases [28]. T3SSs are widely distributed in gram-negative pathogens and are structurally conserved across species. These injectisomes are composed of a basal body that traverses the inner and outer bacterial membrane and a needle-like complex that emerges at its apical end, through which effectors are secreted [29]. The T3SS-1 needle of *S*. Typhimurium is built by the helical polymerization of several hundred subunits of a single small protein (PrgI, 8.9 kDa), while the needle-tip is formed by a pentameric hydrophilic protein complex (SipD, 35.1 kDa) connecting the distal end of the needle to the membrane-spanning translocon (SipB, SipC) [30]. Therefore, during infection, the bacteria receive an external signal from the host environment and begin to assemble coordinately the constituents of the secretion system [31].

Because T3SS-1 is essential for virulence and is conserved among all pathogenic *Salmonella* strains, T3SS-1 proteins appear as ideal candidates for vaccine development. A subunit-based vaccine would provide broader coverage across multiple serotypes and would also simplify vaccine production and formulation.

With this aim, we examined the immunogenicity of the *Salmonella* PrgI and SipD proteins, administered alone or together, by comparing subcutaneous, intranasal and orogastric immunization routes in a mouse model. Protective efficacy was determined against lethal oral intestinal infection with *Salmonella* Typhimurium. We provide the first demonstration that SipD might be a promising target antigen for a *Salmonella* vaccine.

## Methods

### Ethics statement

All experiments were performed in compliance with the French and European regulations on care and protection of laboratory animals (European Community [EC] Directive 86/609, French Law 2001–486, 6 June 2001) and with agreement of the ethical committee (CETEA) no. 12–026 and 15–055 delivered to S. Simon and agreement D-91-272-106 from the Veterinary Inspection Department of Essonne (France).

### Reagents

Biotin N-hydroxysuccinimide ester and streptavidin were from Sigma-Aldrich. Goat anti-mouse IgG and IgM polyclonal antibodies were from Jackson ImmunoResearch. Rat anti-mouse IgG1, IgG2a and IgG2b antibodies were from AbD Serotec. Goat anti-mouse IgA antibodies were from CliniSciences. Sandwich ELISAs were performed with MaxiSorp 96-well microtiter plates (Nunc, Thermoscientific), and all reagents were diluted in Enzyme ImmunoAssay (EIA) buffer (0.1 M phosphate buffer [pH 7.4] containing 0.15 M NaCl, 0.1% bovine serum albumin [BSA], and 0.01% sodium azide). AEBSF (serine protease inhibitor) was from Interchim. Dialysis membranes were from Spectra/Por. Cholera Toxin and Luria Broth were from Sigma. PBS was from Gibco by Life Technologies.

### Recombinant PrgI and SipD production and purification

The prgi and sipd genes of *Salmonella* Typhimurium were synthesized (Genecust) based on the published sequence of strain CIP 104474 (Pasteur Institute Collection), and cloned into *Nde*I/*Xho*I restriction sites of the IPTG inducible pET22b vector (Novagen), allowing insertion of a poly-histidine tag sequence at the 3′ end of the genes. The pET22b recombinant plasmids were used to transform competent *E*. *coli* BL21 (DE3) cells. For each gene, one transformant was grown overnight in Luria Broth (LB) with 100 μg/mL ampicillin at 37°C. 5 mL of this preculture was added to 400 mL of LB + ampicillin for 2 h at 37°C until the mid-log phase was reached (OD_600 nm_ = 0.7) and induced for 3 hours at 37°C by isopropyl-β-D-thiogalactopyranoside (1 mM IPTG) Cells were harvested by centrifugation at 4,000 *x g* for 15 min at 4°C and resuspended on ice in 5 mL of sonication buffer (0.05 M Tris-HCl pH 8, 0.1 M NaCl, 1 mM AEBSF). The bacterial suspensions were then sonicated 3 times at 10–15 Watts for 15 seconds and centrifuged at 14,000 *x g* for 15 min at 4°C. The pellets containing the inclusion bodies were dissolved overnight at 4°C in 5 mL of solubilization buffer (0.05 M Tris-HCl pH 8, 0.2 M NaCl, 8 M urea). After centrifugation at 20,000 *x g* for 10 min at 4°C, the clarified supernatants were diluted with 20 mL of binding buffer supplemented with 10 mM imidazole before application to pre-equilibrated 2 mL of Ni-NTA affinity resin (Chelating Sepharose Fast Flow, GE Healthcare) for 1 h at room temperature. After washing with 20 mL of binding buffer, elution of proteins was performed with elution buffer (0.05 M Tris-HCl pH 8, 0.2 M NaCl, 8 M urea and 0.5 M imidazole). The eluted fractions were pooled and dialyzed in 2 L of 50 mM phosphate buffer, pH 7.4, 150 mM NaCl in a molecular membrane porosity of 3.5 kD. Protein concentrations were measured by absorbance at 280 nm (A_280_) using the NanoDrop Spectrophotometer and the purity was assessed by SDS PAGE (10–15% gradient Phast Gel, Phast system, GE Healthcare). Purified recombinant proteins were stored at -20°C until use.

### Far-UV circular-dichroism (CD)

Far-UV CD spectra were collected for SipD and PrgI. Briefly, a Jasco J-815 spectrometer fitted with a Peltier temperature controller (Jasco) was used to collect spectra from 190 nm to 250 nm through a 0.1-cm-length quartz cuvette. Samples were kept at 20°C and scanned at 100 nm/min with a 1-nm spectral resolution and a 1-s data integration time. All spectra are an average of three measurements. All protein solutions were made to 0.1 mg/mL in potassium phosphate buffer, pH 7.4. Far-UV CD signals were converted to mean residue molar ellipticity.

### Mice and immunizations

Six- to 8-week-old female BALB/c mice (Janvier Labs, France) were used for all experiments, by groups of 14–16 mice. For subcutaneous (SC) and intranasal (IN) immunizations, mice were anesthetized with isoflurane delivered through a vaporizer. Mice were immunized subcutaneously or intranasally on days 0, 21 and 42 with 20 μg of proteins in 100 μL of PBS (SC) or 10 μg in 20 μL of PBS (IN), administered separately or in 1:1 mixture. The proteins admixed with alum hydroxide (1:1) (SC) or with 1.5 μg cholera toxin (IN) adjuvant, were incubated for 1 h in a shaker at room temperature before immunization. For orogastric (OG) immunizations, 300 μg of each protein or of combined proteins (in 200 μL of PBS admixed with 10 μg of cholera toxin) was administered to mice on days 0, 21 and 42 (for the three immunizations (3I) protocol) and on days 0, 21, 42 and 63 (for the four immunizations (4I) protocol). Mice that received only adjuvant and PBS were included as controls. Animals were monitored daily after immunizations.

### LD50 determination and challenge procedures

#### i) LD50 determination

5 mL of preculture of *Salmonella enterica* serovar Typhimurium (*S*. Typhimurium, CIP 104474, Pasteur Institute Collection) was grown in 200 mL of LB at 37°C with agitation (200 rpm) until OD_600 nm_ ~1. Bacteria were centrifuged at 2,000 *xg* for 15 min at 4°C and pellets were resuspended in phosphate-buffered saline (PBS). Serial dilutions were performed in sterile PBS and approximately 2.10^2^ to 2.10^8^ CFU were administered intragastrically (200 μL) to mice (20 to 22 weeks old) using a curved gavage needle (5 mice per group). The exact number of CFU of each challenge dose was recalculated by viable counts (plating serial dilutions on LB agar plates). Mice were monitored twice daily for 25 days. The 50% mouse lethal dose (LD 50) for the challenge strain was calculated by the method of Reed and Muench and determined to be ~10^4^ CFU/mL.

#### ii) Challenge

On day 84 or 105 (for OG 4I) after primary immunization, mice (N = 14–16 per group) were challenged with 100 LD50 of virulent *S*. Typhimurium (~10^6^ CFU/mL, 200 μL in sterile PBS) via the oral route to induce an intestinal infection. Mice were monitored twice daily for 21 days after the challenge and health status, weight and survival were recorded. Any mouse that lost more than 20% of its initial body weight or showed advanced signs of morbidity was euthanized and scored as a death.

### Serum antibody measurement

#### i) Labeling with biotin

100 μg of recombinant protein (PrgI or SipD) in 400 μL of 0.1 M borate buffer pH 8.5 was incubated at a 1:20 molar ratio with biotin-N-hydroxysuccinimide ester dissolved in anhydrous dimethylformamide (DMF). After 30 min at room temperature (RT), the reaction was stopped by adding 100 μL of 1 M Tris-HCl pH 8. Finally, after 30 min at RT, 500 μL of EIA buffer was added and the biotinylated proteins were stored frozen at -20°C until use.

#### ii) Specific IgG and IgA antibodies

Serum IgG antibodies specific for PrgI and SipD were measured by sandwich ELISA. Briefly, microtiter plates were coated with 100 μL of goat anti-mouse Ig(G+M) antibodies or with rat anti-mouse IgG1, IgG2a, IgG2b antibodies at 10μg/mL, and diluted in 50 mM phosphate buffer for 24h at RT. Plates were blocked overnight at 4°C with 300 μL/well of EIA buffer. Serial dilutions of serum samples (from 10^−2^ to 10^−5^) were added in duplicate and incubated overnight at 4°C (100 μL/well). The plates were then washed 3 times before adding 100 μL/well of biotinylated recombinant PrgI or SipD proteins at 100 ng/mL. After 2 hours of incubation at RT followed by three washing cycles, 100 μL/well of acetylcholinesterase (AChE; EC 3.1.1.7)-labeled streptavidin (1 Ellman unit/mL) was added and incubated for 1 hour at RT. Finally, the plates were washed 3 times and the absorbance was measured at 414 nm after 45 min of reaction with 200 μL/well of Ellman’s reagent [32]. Concentrations of Ig(G+M) antibodies and different isotypes were calculated by fitting a calibrated control curve with nonlinear regression and interpolation of absorbance values of test samples by two-phase decay analysis. For measurement of IgA antibodies specific to PrgI and SipD, the titers were measured as described above, using goat anti-mouse IgA antibodies at 2.5 μg/mL for coating. IgA antibody titers were calculated as the reciprocal of the lowest sample dilution giving a signal equal to the average background signal (as measured for 8 wells) plus 10 standard deviations.

### Statistical analysis

All graphics and statistical analysis were generated using GraphPad Prism 5. Statistical significance was assessed using the non-parametric Mann-Whitney test to compare antibody concentrations and titers. Survival curves were compared using a two-tailed Fisher’s exact test.A *P* value <0.05 was considered significant in all determinations.

## Results

### Purification and characterization of recombinant *Salmonella* proteins PrgI and SipD

**Fig 1:**
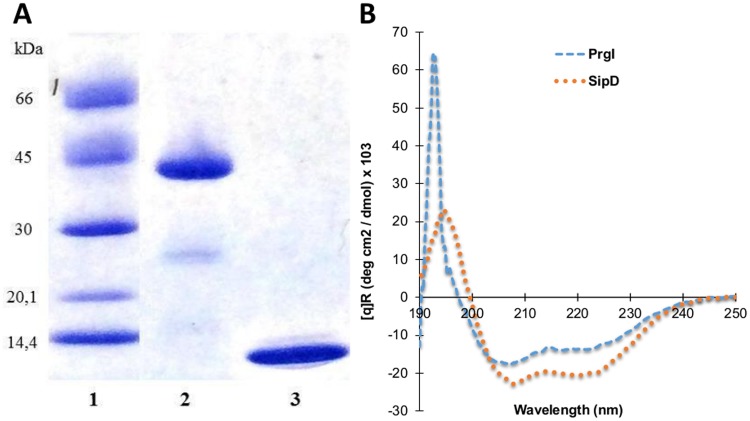
Analysis of recombinant PrgI and SipD proteins. (A) SDS-PAGE / Coomassie blue staining (reducing conditions) of purified recombinant proteins. polyHis-SipD (38.2 kDa, lane 2) and polyHis-PrgI (9.9 kDa, lane 3) are shown with molecular mass markers in kilodaltons (kDa) (lane 1). (B) Far-UV Circular-Dichroism spectroscopy of recombinant proteins were recorded at 20°C in phosphate buffer at pH 7.4.

To produce the large amounts of purified PrgI and SipD proteins necessary to immunize mice, the corresponding genes were cloned into the IPTG inducible pET22b plasmid, generating genes carrying a poly-histidine tag sequence at their 3’ ends. The resulting recombinant vectors were then introduced into *E*. *coli* BL21 and expression of the proteins led to the production of 2.3 mg/L and 1.4 mg/L of SipD and PrgI, respectively, in inclusion bodies. Purity of the proteins was assessed by SDS PAGE electrophoresis and Coomassie blue staining (Fig 1A). Far-UV CD spectroscopy was employed to assess the secondary structure of the purified recombinant proteins. The CD measurements of PrgI and SipD showed spectra exhibiting dominant minima at 208 and 222 nm, characteristic of proteins with α-helical secondary structures (Fig 1B) thus assessing their correct refolding.

### Immunizations with PrgI and/or SipD proteins induce Ig(G+M) antibody responses

**Fig 2:**
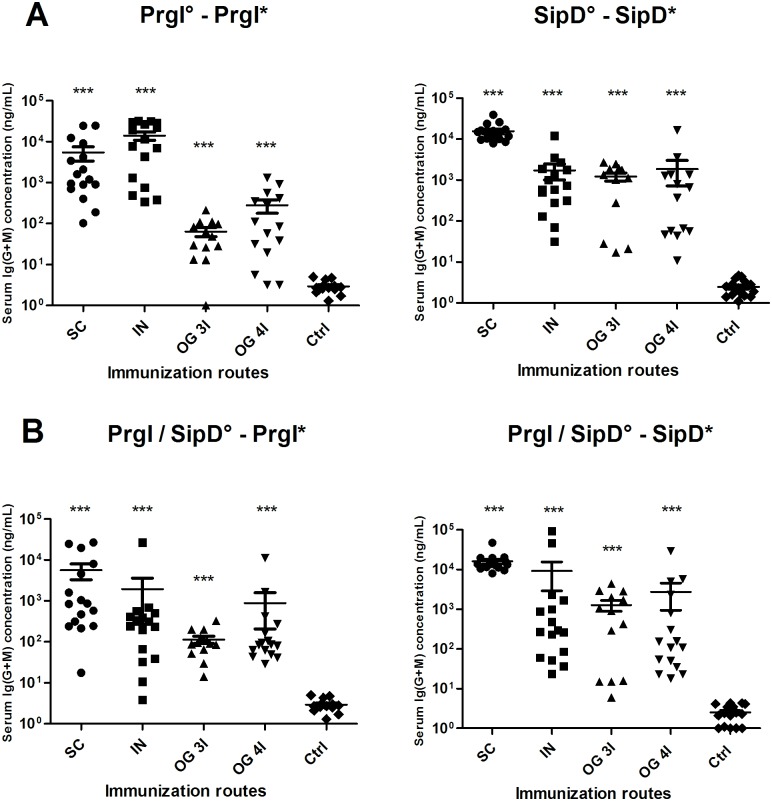
Serum Ig(G+M) concentrations of mice immunized with PrgI or SipD (A) and PrgI/SipD (B). Serum Ig(G+M) antibodies specific for PrgI (left) and SipD (right) were quantified by sandwich ELISA 2 weeks after the last immunization as described in Materials and Methods. Data represent mean concentrations (ng/mL) and the standard errors (SEM) from 14–16 individual mice per group. Asterisks *** indicate *P* value< 0.001, comparing the antibody responses using different routes versus control mice. No cross-reactions were observed between PrgI and SipD (data not shown). [°: indicates injected immunogen; *: indicates biotinylated recombinant protein].

The kinetics of serum Ig(G+M) responses against *Salmonella* PrgI and SipD are shown in S1 Fig. Mice immunized subcutaneously (SC), intranasally (IN) and orally (OG) with PrgI and SipD proteins separately (Fig 2A) or combined (Fig 2B) in the presence of alum (SC) or cholera toxin (CT, for IN and OG immunizations) developed antigen-specific antibody responses. Except for IN immunization, whatever the other routes of immunization, the specific antibody titers against each protein were equivalent in terms of concentrations and kinetics when proteins were administered alone (PrgI or SipD) or together (PrgI/SipD) (compare panels A and B for each route of immunization), meaning that none of the proteins in the mixed administration was dominant over the other and used the immune response to its advantage. Except for the IN route, the antibody titers with SipD were greater than with PrgI (Table 1). For all immunization routes, serum Ig(G+M) antibodies to SipD and PrgI were detected rapidly (2 weeks) after the first immunization and reached a plateau after the second (SipD, SC route), the third (IN routes for PrgI and SipD, SC for PrgI) or the fourth immunization (OG routes, PrgI and SipD). PrgI-specific Ig(G+M) concentrations reached the highest values by the IN route (14 μg/mL measured at day 84, one month after the third immunization, see Table 1) up to 2 logs better than OG or SC routes. Comparatively, SipD-specific Ig(G+M) production after IN immunization was delayed, and the peak concentration was ten-fold reduced compared with PrgI-specific Ig(G+M) (1.7.10^3^ ng/mL at day 84). In contrast, titers obtained for SipD immunizations were one log better by the SC route than the others.

**Table 1:** Summary of the antibody responses (IgG and IgA) after the last immunization with PrgI or SipD by the SC, IN and OG routes.

Immunization route	Immunogen	Ig(G+M) ng/mL	IgG 1 ng/mL	IgG (2a+2b) ng/mL	IgA titers
**SC**	PrgI	5.4.10^3^	1.4.10^4^	1.10^4^	
	SipD	1.5.10^4^	1.3.10^4^	3.5.10^2^	
**IN**	PrgI	1.4.10^4^	1.6.10^3^	4.2.10^3^	6.1.10^2^
	SipD	1.7.10^3^	2.6.10^3^	4.65.10^2^	2.4.10^2^
**OG 3I**	PrgI	6.4.10^1^	1.15.10^2^	6.8.10^1^	6.8.10^2^
	SipD	1.3.10^3^	6.8.10^3^	9.9.10^2^	1.6.10^3^
**OG 4I**	PrgI	2.9.10^2^	1.5.10^3^	1.6.10^2^	4.3.10^2^
	SipD	1.8.10^3^	5.2.10^3^	3.2.10^3^	7.9.10^2^

Data represent mean concentrations (ng/mL) with SEM for IgG responses and mean titers with SEM for IgA responses from each group of mice.

Because entry of pathogenic *Salmonella* occurs via the oral route, oral immunization would induce a first line of defense at the mucosal epithelial surface, through inhibition of bacterial penetration into the PPs. With this goal in mind, we examined the immunogenicity of PrgI and SipD when administered to mice orally in the presence of CT as adjuvant. Two studies were performed in which we compared the effect of 3 or 4 injections of the proteins. The groups immunized with SipD, in both cases, exhibited the highest levels of serum Ig(G+M) with no significant difference between the 2 protocols (1.3 vs 1.8 μg/mL, 4 weeks after the last immunization). In contrast, the mice immunized with PrgI, responded poorly in both cases (290 ng/mL, after the last immunization), confirming that PrgI was less immunogenic than SipD.

### Intranasal and orogastric administrations of PrgI and SipD elicit serum IgA titers

**Fig 3:**
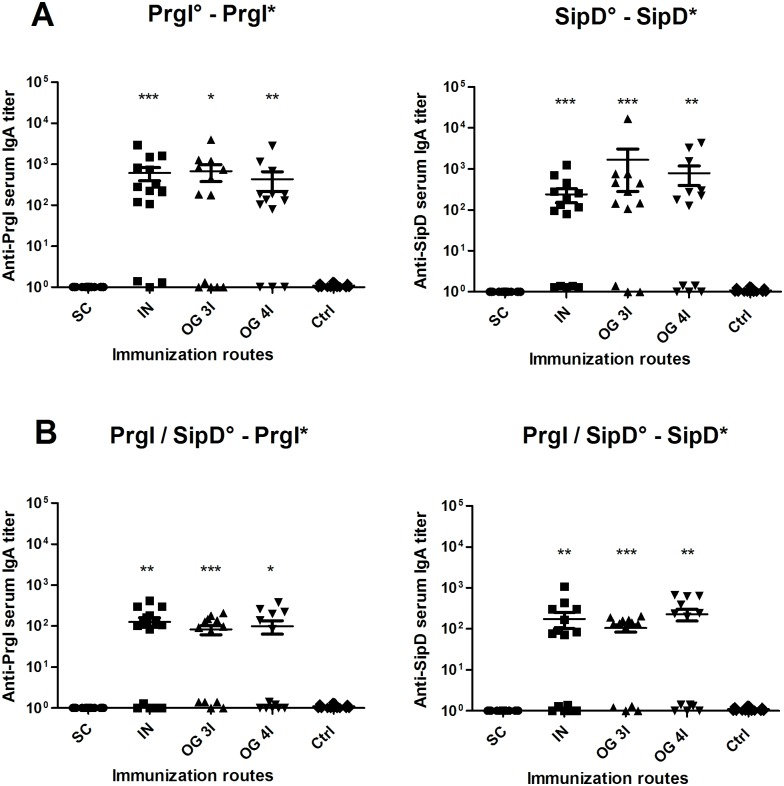
IgA titers of mice immunized with PrgI or SipD (A) or PrgI/SipD (B). Serum IgA antibodies specific for PrgI (left) and SipD (right) were measured by sandwich ELISA 2 weeks after the last immunization as described in Materials and Methods. Data represent mean titers and the standard errors (SEM) from 14–16 individual mice per group. Asterisks indicate *P* values: *** *p* < 0.001, ** 0.001<*p <*0.01 and * *p*< 0.05 when comparing mice immunized by the IN or OG route versus mice immunized by the SC route and control mice. No cross-reactions were observed between PrgI and SipD (data not shown). [°: indicates injected immunogen; *: indicates biotinylated recombinant protein].

To evaluate the induction of IgA antibodies in the mucosa, which represent the first line of adaptive immune defense against enteric pathogens, the PrgI- and SipD-specific IgA titers in serum from immunized and control mice were measured, 2 weeks after the last immunization (Fig 3 and Table 1). As expected, and like controls, mice immunized subcutaneously did not produce any IgA antibodies. For each protein, the specific IgA titers were equivalent for mice immunized intranasally or orogastrically 3I or 4I (Table 1). The titers of PrgI-specific IgA in mice immunized by the IN route were slightly higher than those of mice immunized IN with SipD, as observed for IgG titers. Comparatively, the IgA titers obtained for mice immunized with both proteins were lower than those obtained for mice immunized by each protein separately (Tables 1 and 2).

**Table 2:** Summary of the antibody responses (IgG and IgA) after the last immunization with both proteins (PrgI and SipD) by the SC, IN and OG routes.

Immunization route	Immunoge PrgI / SipD	Ig(G+M) ng/mL	IgG 1 ng/mL	IgG (2a+2b) ng/mL	IgA titers
**SC**	PrgI*	5.7.10^3^	6.7.10^3^	4.3.10^2^	
	SipD*	1.5.10^4^	5.7.10^3^	2.7.10^2^	
**IN**	PrgI*	1.9.10^3^	4.9.10^1^	9.5.10^2^	1.2.10^2^
	SipD*	9.2.10^3^	1.10^3^	4.5.10^2^	1.7.10^2^
**OG 3I**	PrgI*	1.2.10^2^	7.10^1^	1.2.10^2^	8.10^1^
	SipD*	1.2.10^3^	2.10^3^	6.10^2^	1.6.10^2^
**OG 4I**	PrgI*	9.10^2^	1.10^2^	2.10^2^	8.4.10^1^
	SipD*	2.7.10^3^	1.2.10^3^	3.4.10^2^	2.3.10^2^

Data represent mean concentrations (ng/mL) with SEM for IgG responses and mean titers with SEM for IgA responses from each group of mice (*: indicates biotinylated recombinant protein).

### Immune response involved all main IgG isotypes in serum

**Fig 4:**
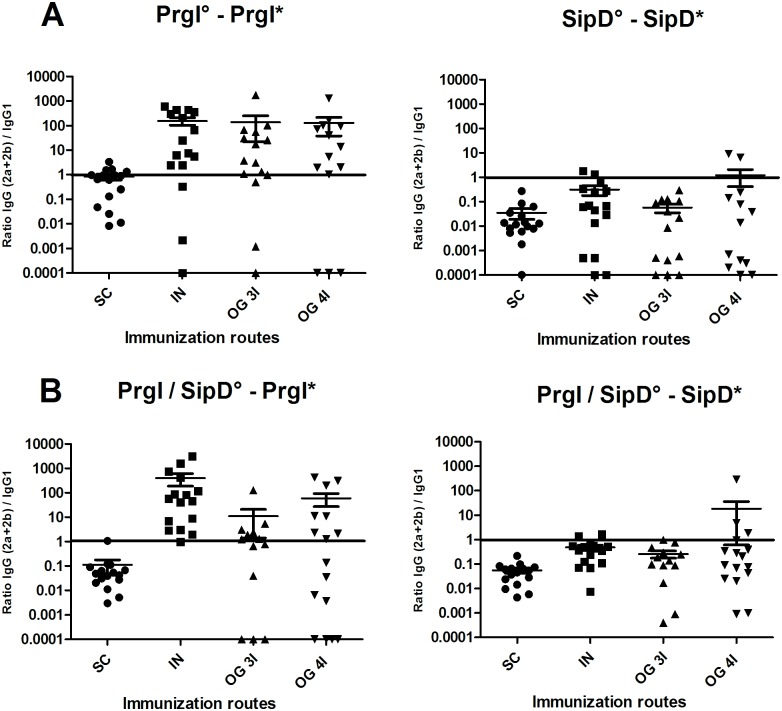
IgG (2a +2b) / IgG 1 ratio after PrgI (left) and SipD (right) immunizations. Mice immunized with PrgI or SipD separately are represented on panel A and those receiving both PrgI and SipD on panel B. Data represent mean and the standard errors (SEM) from 14–16 mice per group. [°: indicates immunogen injected; *: indicates biotinylated recombinant protein].

To investigate further the immune response elicited by the different routes of immunization, the PrgI- and SipD-specific IgG1, IgG2a and IgG2b subclasses were measured in serum from immunized and control mice at day 56 (after the third immunization) for the SC, IN, OG (3I) routes and at day 77 (after the fourth immunization) for the OG (4I) route (S2 Fig) and Table 1). Measurement of the IgG isotype concentrations in sera of immunized mice revealed that all main subclasses contributed to the humoral response whatever the route. It should be noted that for the majority of Ig(G+M) measurements (Table 1), the concentrations were below the sum of the concentrations obtained for the different IgG isotypes. This could be due to the antibodies used for the standard curve in the sandwich ELISA: a mixture of specific PrgI or SipD IgG1:IgG2a:IgG2b (1:1:1) was used as a standard of Ig(G+M) polyclonal antibodies, which does not exactly reflect the diversity of a polyclonal response (and particularly the IgM production), by comparison with the other tests where each specific isotype was used. Overall, IgG1 were found in higher concentration after SC route immunization compared with the other routes, for PrgI or SipD. While SipD elicited a strong IgG1 response whatever the route, PrgI IgG1 quantities were much lower (10- to 100-fold) by the IN and OG routes compared with the SC route (upper panels A and B, S2 Fig and Table 1). The same profile was obtained for specific PrgI IgG1 and IgG(2a+2b) antibodies, with the highest concentration (14 μg/mL) obtained for the SC route and the lowest (100 ng/mL) for the OG (3I) route. IgG1 and IgG(2a+2b) are respectively indicators of the T helper type 2 (humoral) and type 1 (cellular) immune responses. IgG(2a+2b):IgG1 ratios were taken as indicators of the T helper type 1 (Th1) / Th2 balance, in order to evaluate the contribution of each pathway to the immune response. As *Salmonella* are facultative intracellular pathogens and multiply in macrophages, the ratio of IgG(2a+2b) to IgG1 titers was determined (Fig 4). For PrgI immunizations, the Th1/Th2 balance was clearly in favor of the cellular (Th1) immune response (100-fold more IgG(2a+2b) than IgG1) for the IN and OG routes, and close to 1 for the SC route (Fig 4). Interestingly, the profile of SipD-specific antibodies was the opposite of the PrgI antibody profile: a 10-fold higher IgG1 response was obtained for the OG route (5 to 7 μg/mL) than IgG(2a+2b) antibody response (S2 Fig and Table 1). Thus, the Th1/Th2 balance appears in favor of humoral immunity for the SipD immunogen (Fig 4). Similar results were obtained when both proteins were administered together, with a Th1/PrgI and a Th2/SipD response (compare left panels A and B for PrgI and right panels A and B for SipD, S2 Fig and Fig 4).

### Protective efficacy against lethal *S*. Typhimurium oral challenge

**Fig 5:**
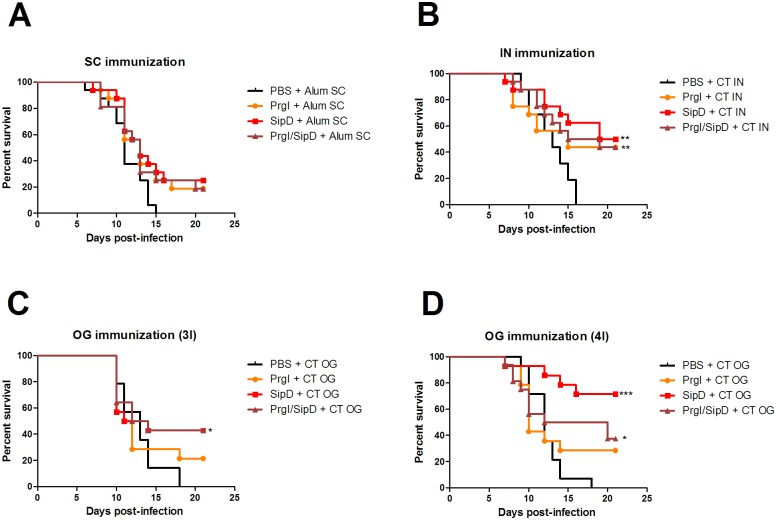
Protective efficacy of PrgI and SipD (A-D). Mice (N = 14–16) were immunized at days 0, 21 and 42 or 0, 21, 42 and 63 by the indicated routes. Six weeks after the last immunization: at day 84 for the SC (A), IN (B) and OG 3I (C) routes; at day 105 for the OG 4I (D) route, 10^6^ CFU/mL (100 LD 50) of *S*. Typhimurium were administered orally to immunized and control mice. Survival was monitored for 21 days. Statistical significance was determined using a 2-tailed Fisher's exact test. Statistically significant differences are indicated by *** *p* < 0.001, ** 0.001<*p <*0.01 and * *p*< 0.05 compared to PBS groups.

The oral lethal dose 50% (LD50) of the *S*. Typhimurium strain used in the experiments (see experimental procedures) was determined at 10^4^ CFU/mL. To assess the protective efficacy induced by PrgI or SipD, immunized and control mice were subjected to oral challenge, six weeks after the last immunization, with ~100 LD50 (10^6^ CFU/mL) of *S*. Typhimurium (Fig 5A–5D). In all challenges, the mortality rate of control animals (PBS/adjuvant immunized mice) was 100% with death occurring at 15–18 days after challenge. The protective efficacy of the PrgI and SipD proteins by SC immunization was 19% and 25%, respectively (Table 3). Two-fold higher protection was observed for mice vaccinated by the IN route (44% and 50% for PrgI and SipD, respectively). Mice immunized thrice orally with PrgI and SipD did not exhibit better protection (21.5% and 43%, respectively). The highest level of protection (71.5%) was obtained for mice immunized four times by the oral route with SipD, while those immunized with PrgI exhibited only 29% protection. In all cases, the admixed proteins provide less or equivalent protection than SipD alone, showing that there was no synergistic protection effect of proteins administered together.

**Table 3:** Protection efficacy of PrgI and SipD T3SS proteins in mice from lethal challenge with *S*. Typhimurium.

Immunization route	Immunogen	Mortality (no. of dead mice / total no. of mice)	*P* value^a^	Protection efficacy (%)
**SC**	PrgI	13/16	0.226	19
	SipD	12/16	0.101	25
	PrgI / SipD	13/16	0.226	19
**IN**	PrgI	9/16	0.007	44
	SipD	8/16	0.002	50
	PrgI / SipD	9/16	0.007	44
**OG 3I**	PrgI	11/14	0.222	21.5
	SipD	8/14	0.016	43
	PrgI / SipD	8/14	0.016	43
**OG 4I**	PrgI	10/14	0.098	29
	SipD	4/14	< 0.0001	71.5
	PrgI / SipD	10/16	0.019	37.5
**Control**	PBS	16/16		

The mice were challenged with 10^6^ CFU/mL of *S*. Typhimurium by the oral route (LD 50 = 10^4^ CFU/mL).

^a^ The mortality rate of the immunized group was compared with that of the PBS-immunized control animals using the 2-tailed Fisher’s exact test.

## Discussion

The development of vaccines against *Salmonella* is very challenging because of the multiplicity of serotypes and the ability of the bacteria to live both extra- and intracellularly [33, 34].

There are currently two commercially available vaccines (Ty21a live attenuated and Vi capsular polysaccharide). Both are protective only against enteric (typhoidal) fever and do not cover non-typhoidal diseases. Moreover, they are less effective in immunocompromised adults or children under 2 years of age, for whom the invasive *Salmonella* diseases represent the highest burden [35–37], and the increase of iNTS multidrug resistance threatens treatment outcomes even more. Overcoming these obstacles is challenging and still a matter of great concern [13, 18]. With this in mind, we focused the criteria of selection of a good vaccine candidate on: i) a conserved antigen among the different *Salmonella* serotypes, allowing broad-spectrum coverage, ii) a soluble antigen, which is easier and cheaper to produce and refold than membrane proteins, and iii) an immunogen avoiding reactogenicity and allowing protection in children under 2 years of age. Among potential antigens, we focused our attention on two proteins of the *Salmonella* type III secretion systems (T3SS). Salmonellae encode two T3SS necessary for the virulence of the bacteria and which appear to be involved in two crucial steps of the pathogenesis: invasion of epithelial cells and survival in macrophages [23, 38]. Until recently, the role attributed to each of the T3SS was sequential: T3SS-1 was expressed first and appeared responsible for invasion of non-phagocytic cells, leading to acute local disease (intestinal inflammation and diarrhea), while T3SS-2 was expressed secondarily and was essential for survival and replication in macrophages, thus playing a major role in dissemination of the bacteria and systemic disease. It appears now that T3SS-2 might be expressed earlier, even before intestinal penetration of the bacteria [27], and that T3SS-1 might be expressed also at later stages and that some of its effectors persist within host cells long after internalization of the bacteria. In this regard, some effectors of T3SS-1 have been shown for the first time to play a role in systemic infection in mice [39]. Moreover, both systems could cooperate in intracellular replication of the bacteria [23]. Vaccines against extracellular bacteria engage humoral immunity, while those targeting intracellular bacteria necessitate the stimulation of cellular immunity to destroy infected macrophages [33, 34].

In order to evaluate the role of T3SS-1 in the virulence of *Salmonella* and with the aim of developing a safe and effective vaccine to prevent iNTS disease, we decided to investigate the protective efficacy of two conserved proteins of T3SS-1 that could provide broad protection against multiple *Salmonella* serotypes. Partly inspired by what has already been published on IpaD, the SipD *Shigella* counterpart, for which protective efficacy against *Shigella* challenge was observed [40], we explored the protective capacity of PrgI and SipD proteins using different immunization routes with an approved set of adjuvants in a murine model of oral *S*. Typhimurium infection. PrgI composes the needle that allows the passage of effector proteins into the cytoplasm of the host cells, and SipD localizes to the needle tip, where it controls further assembly of the T3SS as well as T3SS effector secretion [29]. For the first time, we have demonstrated that one T3SS composing protein is able to induce very good levels of protection (up to 72% with SipD, by the oral route) against *S*. Typhimurium using a lethal intestinal challenge murine model. We decided to focus on humoral response and have tried to correlate the levels of protection with the levels of production of specific antibodies. We have shown that, except for the IN route, the levels of specific antibodies directed to SipD were systematically superior to those of PrgI, suggesting that SipD was more immunogenic than PrgI. Despite the very high Ig(G+M) titers obtained by the SC route of immunization, protection was poor and less than by the other routes (IN and OG), for both proteins.

Regarding PrgI, the protein composing the needle of the T3SS syringe, the best protection was obtained by the IN immunization route, which elicits the highest quantity of antibodies. However, this protection reached a maximum of 40% and the Th1/Th2 balance suggests that this protein induces not only humoral but also cellular immunity. Immunization by the oral route was unfavorable for PrgI (21% protection) and increasing the immunizations had no impact on protective efficacy. Although naturally polymerizing in multimers, the small size of the elementary subunit (9 kDa) probably played a role in gastric degradation. In contrast, SipD, the protein of the injectisome needle tip, elicited a strong humoral systemic (as suggested by the Th1/Th2 balance in favor of Th2 response) and mucosal immunity by the OG route and subsequent efficient protection against infection (72% protection with four immunizations). These results highlight the importance of mucosal immunity for protection against *Salmonella* infection already described as the first line of defense before dissemination of the pathogen [41]. Although many studies have already demonstrated the importance of cellular immunity in the control of intracellular *Salmonella* infection [42], there is also converging evidence of the importance of humoral immunity in the fight against *Salmonella* [34, 43–45]. Evidence from humans studies suggest that severe infection and bacteremia occur when specific antibody is lacking, whose role is essential to protect against extracellular growth of NTS in the blood [46, 47]. As an example *Salmonella*-specific antibody responses in young African children are associated with resistance to invasive NTS disease [44]. Until now immune targets in human Salmonellosis have been poorly characterized. Different techniques including proteomic microarrays or immunoaffinity coupled to proteomic analysis have been developed and provide a comprehensive overview of immune response using sera from infected patients [48, 49]. From these studies, different antigens either from *S*. Typhi or NTS have been characterized as potential targets for vaccine development (OmpA, flagellin, SseB (T3SS-2 effector protein), PhoN as examples). None of the T3SS-1 proteins have been characterized by those techniques. However given the major role of T3SS-1 in virulence of other closely related facultative intracellular bacteria (*Shigella*), and immune response against IpaD generated by *Shigella* infection [50] one could expect that SPI-1 T3SS effector proteins might be good antigens for vaccine development.

Although sustained efforts have been made in the development of live attenuated vaccines of inactivated whole cells against typhoidal and non-typhoidal *Salmonella* diseases, few studies describe the protective effects of protein-based vaccines [51], and were mainly focused on porins, flagellin and polysaccharides [52–54]. To our knowledge, ours is the first study describing a protective effect of a T3SS-1 component. The role of T3SS-1 and particularly SipD effector in systemic dissemination of the bacteria [39] strengthens the protective effect obtained using SipD as immunogen and underscores the importance of the extracellular life cycle of the bacterium for its pathogenicity and dissemination. The molecular mechanisms governing the protection induced by SipD remain to be deciphered in the light of the protective effect already described for T3SS IpaD effector [55]. The novelty of the results obtained in this study should highlight the major role of SPI-1 T3SS in *Salmonellae* virulence and although giving first evidence of the interest of SipD as a potential target to protect against *Salmonella* infection in development of new vaccines, this study also presents limitations that should be overcome in the future in order to progress on the road of a potential human application. As examples, production of the recombinant protein should necessitate fine tuning (precise refolding control, assessment of high purity degree of the protein, suppression of the His-tag …) and although CT remains one of the most potent known mucosal adjuvants, it suffers from high toxicity. It would be necessary to evaluate other less toxic mucosal immunoadjuvants and to reduce, if possible, the number of immunizations. On the other hand, because of the key role of SipD in the virulence of the bacteria, it is well conserved among the different *Salmonella* strains and species (between 80% and 85% protein sequences identity, Table 4) and thus appears as a good target for broad-spectrum coverage against different *Salmonella* species and serotypes. However, as there are over 2000 serovars of *Salmonella* that can infect humans, further investigations are needed to evaluate the possibility of a broad-spectrum coverage (for example against another relevant serovar *S*. Enteritidis) as well as the value of associating different targets with SipD (porins, T3SS-2 molecules as SSeB, shown to reduce the bacterial load in a murine model of *S*. Typhimurium infection [56], polysaccharides…).

**Table 4:** SipD protein identity sequences for the 4 major *Salmonella* pathogens of humans.

*Salmonella* spp.	SipD identity (% of *S*. Typhimurium sequence)	ID sequence number NCBI ID
***S*. Typhimurium**	100%	AAA86617.1
***S*. Enteritidis**	85%	CID63174.1
***S*. Typhi**	81%	CAA57990.1
***S*. Paratyphi A**	85%	CDU43169.1

### Accession numbers

The ID numbers of proteins mentioned in the text are AAB60189.1 for PrgI and AAA86617.1 for SipD (from NCBI).

## Supporting Information

S1 Fig Kinetics of serum Ig(G+M) responses to PrgI and SipD antigens. Mice were immunized three or four times (indicated with arrows) with PrgI or SipD as described in Materials and Methods. Serum Ig(G+M) antibodies specific for PrgI (left) and SipD (right) were quantified by sandwich ELISA. Data represent mean concentrations (ng/mL) and the standard errors (SEM) from 14–16 individual mice per group. *P* value < 0.001, comparing the antibody responses on days post-immunization versus those on day 0. No cross-reactions were observed between PrgI and SipD (data not shown). [°: indicates injected immunogen; *: indicates biotinylated recombinant protein].
(TIF)

S2 Fig Kinetics of serum Ig(G+M) responses to PrgI and SipD antigens. Mice were immunized three or four times (indicated with arrows) with PrgI and SipD as described in Materials and Methods. Serum Ig(G+M) antibodies specific for PrgI (left) and SipD (right) were quantified by sandwich ELISA. Data represent mean concentrations (ng/mL) and the standard errors (SEM) from 14–16 individual mice per group. *P* value < 0.001, comparing the antibody responses on days post-immunization versus those on day 0. No cross-reactions were observed between PrgI and SipD (data not shown). [°: indicates injected immunogen; *: indicates biotinylated recombinant protein].
(TIF)

S3 Fig Concentrations of Ig mouse isotypes. Serum IgG1, IgG2a and IgG2b subclasses specific for PrgI (A, B left) and SipD (A, B right) were quantified by sandwich ELISA, 2 weeks after the last immunization. Mice immunized with PrgI or SipD separately are represented on panel A and those receiving PrgI / SipD together on panel B. Data represent mean concentrations (ng/mL) and the standard errors (SEM) from 14–16 mice per group. Asterisks indicate *P* values: *** *p* < 0.001 and ** 0.001<*p <*0.01 when comparing immunized mice versus control mice. No cross-reactions were observed between PrgI and SipD (data not shown). [°: indicates immunogen injected; *: indicates biotinylated recombinant protein].
(TIF)
