# Supplementary figures and images for: Role of T3SS-1 SipD Protein in Protecting Mice against Non-typhoidal Salmonella Typhimurium
Source: PLoS Negl Trop Dis. 2016 Dec 19;10(12):e0005207. doi: 10.1371/journal.pntd.0005207 (PMC5167260; doi:10.1371/journal.pntd.0005207)

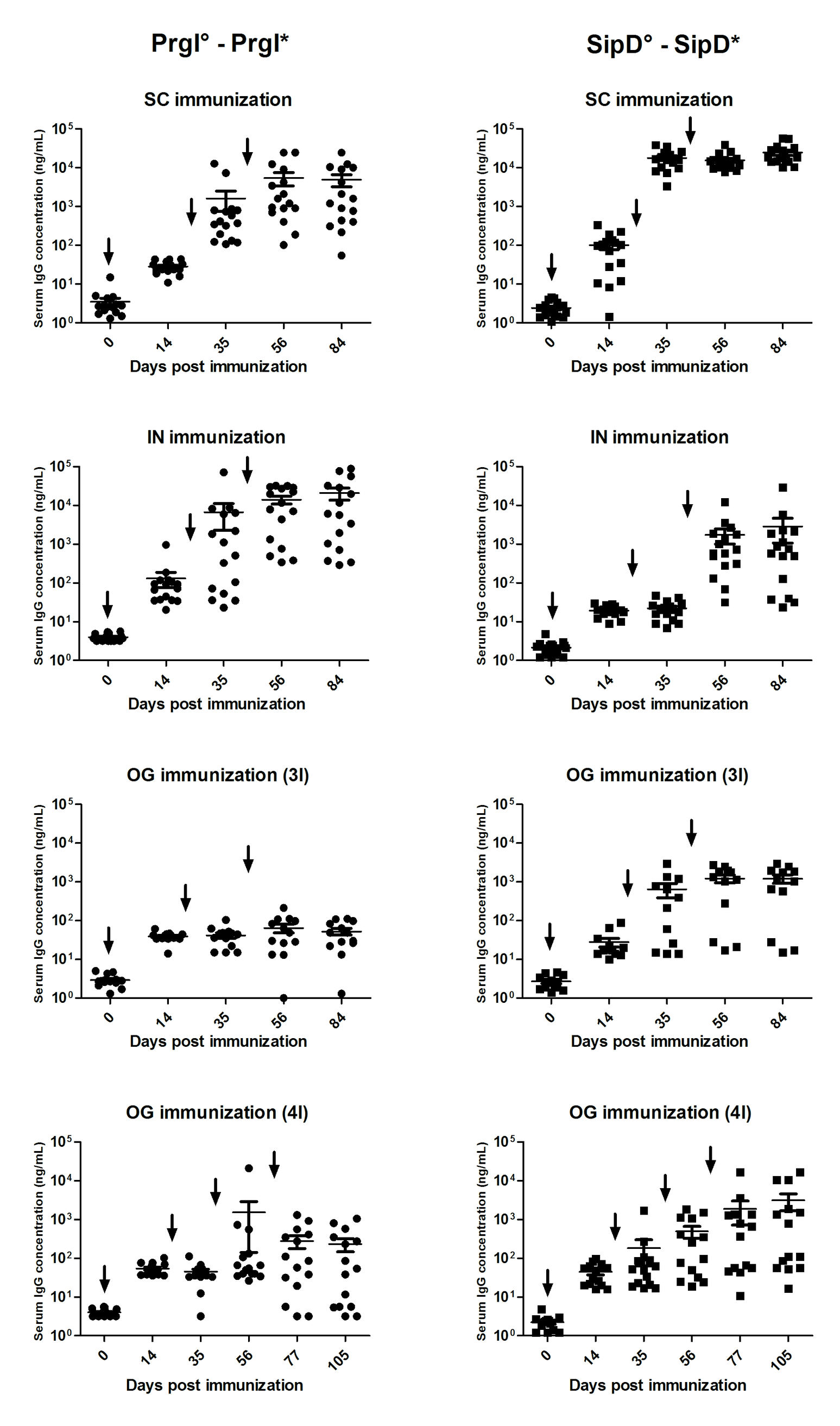

Supplement: S1 Fig — Mice were immunized three or four times (indicated with arrows) with PrgI or SipD as described in Materials and Methods. Serum Ig(G+M) antibodies specific for PrgI (left) and SipD (right) were quantified by sandwich ELISA. Data represent mean concentrations (ng/mL) and the standard errors (SEM) from 14–16 individual mice per group. P value < 0.001, comparing the antibody responses on days post-immunization versus those on day 0. No cross-reactions were observed between PrgI and SipD (data not shown). [°: indicates injected immunogen; *: indicates biotinylated recombinant protein]. (TIF) [file pntd.0005207.s001.tif]

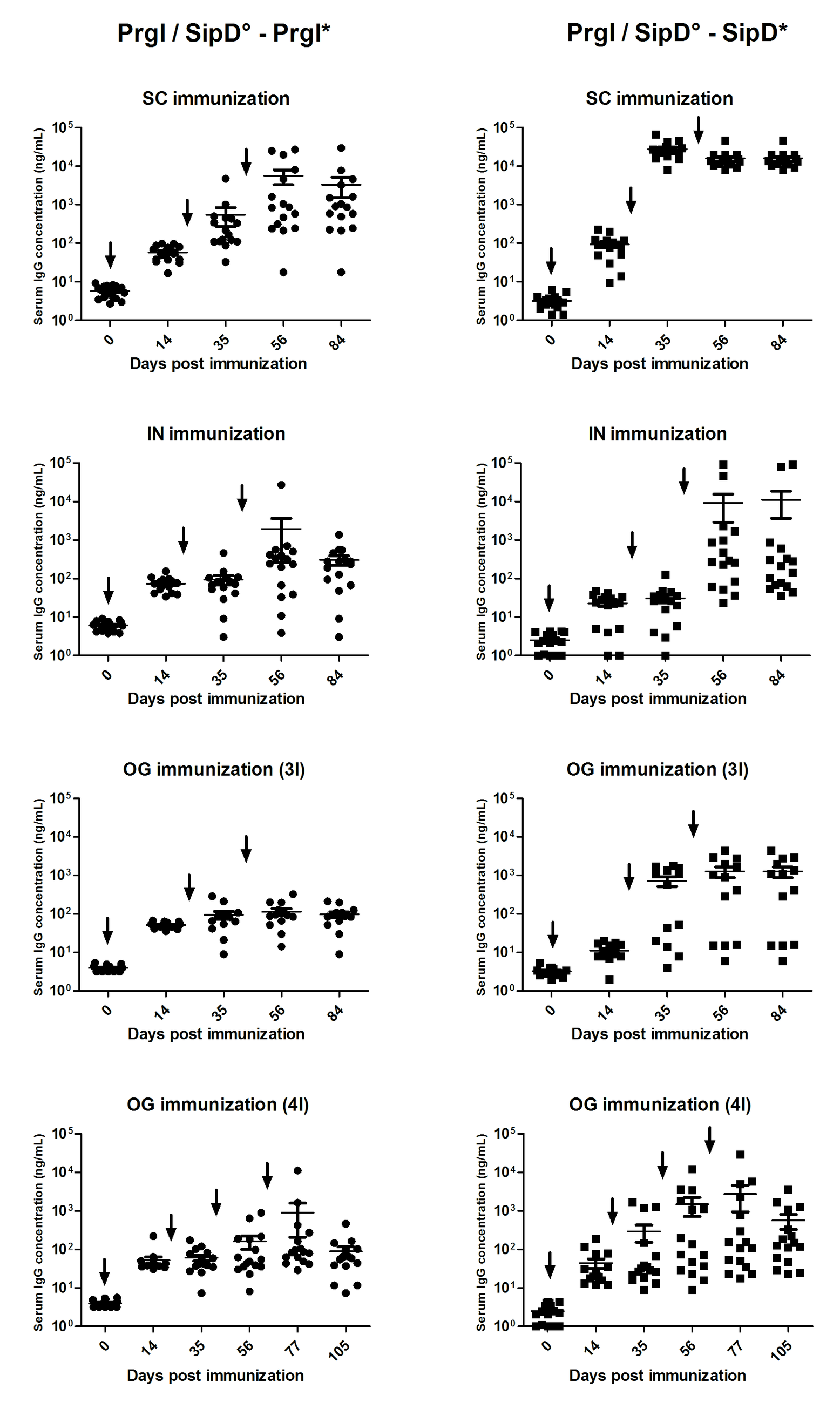

Supplement: S2 Fig — Mice were immunized three or four times (indicated with arrows) with PrgI and SipD as described in Materials and Methods. Serum Ig(G+M) antibodies specific for PrgI (left) and SipD (right) were quantified by sandwich ELISA. Data represent mean concentrations (ng/mL) and the standard errors (SEM) from 14–16 individual mice per group. P value < 0.001, comparing the antibody responses on days post-immunization versus those on day 0. No cross-reactions were observed between PrgI and SipD (data not shown). [°: indicates injected immunogen; *: indicates biotinylated recombinant protein]. (TIF) [file pntd.0005207.s002.tif]

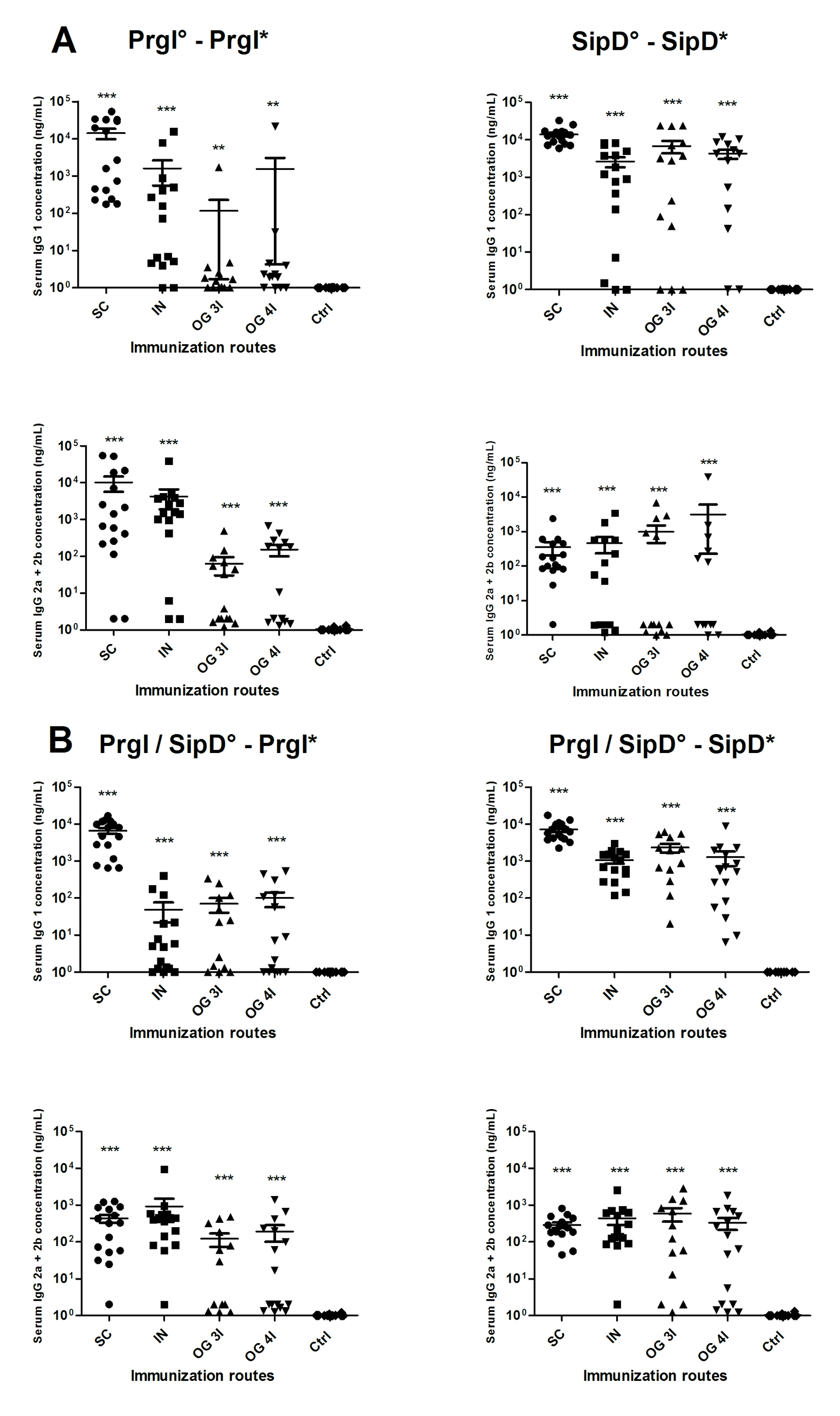

Supplement: S3 Fig — Serum IgG1, IgG2a and IgG2b subclasses specific for PrgI (A, B left) and SipD (A, B right) were quantified by sandwich ELISA, 2 weeks after the last immunization. Mice immunized with PrgI or SipD separately are represented on panel A and those receiving PrgI / SipD together on panel B. Data represent mean concentrations (ng/mL) and the standard errors (SEM) from 14–16 mice per group. Asterisks indicate P values: *** p < 0.001 and ** 0.001<p <0.01 when comparing immunized mice versus control mice. No cross-reactions were observed between PrgI and SipD (data not shown). [°: indicates immunogen injected; *: indicates biotinylated recombinant protein]. (TIF) [file pntd.0005207.s003.tif]
